# Review of Nature-based Solutions in Dryland Ecosystems: the Aral Sea Case Study

**DOI:** 10.1007/s00267-023-01822-z

**Published:** 2023-04-28

**Authors:** Shahzoda Alikhanova, Joseph William Bull

**Affiliations:** Department of Biology, University of Oxford, 11a Mansfield Road, OX1 3SZ Oxford, UK

**Keywords:** Nature-based solutions (NbS), Drylands, Aral Sea, Uzbekistan

## Abstract

NbS have gained substantial attention in the academic literature recently as a potential approach for simultaneously tackling environmental issues and addressing societal challenges. Drylands, which are among the world’s most vulnerable areas to the impacts of climate change and cover a little less than the half of the global terrestrial surface, were the focus of this study. We conducted a systematic literature review to explore the potential opportunities for the application of NbS in rural drylands across the globe. We go on to specifically consider the possibility of applying selected NbS approaches in the Aral Sea region of Uzbekistan, as a case study of a dryland ecosystem illustrating major environmental and social challenges. We highlight which NbS show the most promise in the Aral Sea region and conclude with a discussion of existing gaps in the literature on NbS in drylands, and opportunities for further research.

## Introduction

Drylands cover over 45% of the global terrestrial area, and are home to nearly one-third of the global human population, 90% of which reside in developing countries (UNCCD, 2017), (IUCN, 2017), (Prăvălie, 2016). According to Food and Agriculture Organisation of the United Nations (FAO), drylands globally comprise the following categories: barren land (28%), grassland (25%), forests (18%), and cropland (14%); with the remainder classified as “other land” (which includes inland water bodies, other wooded land, apart from those classified as forests, built-up environment and unidentified land types) (FAO, 2019). Drylands are rich in biodiversity and contain rare and endemic species that are not present in other biomes (IUCN, 2017). Dry areas are reported to be among the most vulnerable to land degradation caused by climate change and anthropogenic activities (UNCCD, 2017), and, in case of the global temperature rise over targeted 1,5 °C, they will be more susceptible to increased surface warming than humid areas. This would in turn mean increased vulnerability to droughts, decreased water availability, reduced crop yields and increased disease transmission (e.g., malaria) (Huang, Yu, Dai, Wei, & Kang, 2017), (UNCCD, 2017). Reports also state that areas covered by drylands are expected to substantially expand by the end of the 21st century reaching almost 60% of the global land coverage (UNCCD, 2017), (Prăvălie, 2016), (Feng & Fu, 2013).

Nearly half of the Asian continent is covered by drylands, which contributes to an estimated 34% of the world’s drylands. Dryland areas have largely expanded in Asia since mid-twentieth century stretching towards north latitudes, triggering water stress and limiting ecosystem services (Prăvălie, 2016), (Yao, Fu, Liu, Wang, & Song, 2021). Central Asia, which contains extensive dryland ecosystems, is among the regions most heavily impacted by the global climate change, and the trends are projected to accelerate in the coming decades (Lioubimtseva & Henebry, 2009), (Yushanjiang, Zhang, & Leong Tan, 2021), (Schlüter, et al., 2013), (Yang, et al., 2019) putting even more pressure on precious natural resources and already fragile ecosystems. In the long term, populations of Central Asian countries are likely to suffer from the severe consequences of land degradation, desertification and food security issues imposed by climate change if no immediate actions for adaptation and mitigation are taken.

The desiccation and subsequent almost complete loss of the Aral Sea, once the world’s fourth largest inland water body (Lemly, Kingsford, & Thompson, 2000), having had far-reaching social, economic and ecological implications in all countries of Central Asia aggravated the situation even more. According to reports, Central Asia and Uzbekistan in particular, is among the areas most effected by dust and salt storms due to soil and wind erosion (Li, Ma, & Zhang, 2020) with an alarming number of people with respiratory diseases caused by these natural events (Li, Ma, & Zhang, 2020). Nature-based solutions, as a recently emerged approach for sustainably mitigating and adapting to environmental challenges, have gained considerable attention in the scientific literature recently. They have been seen as a sustainable tool for the provision of benefits both for the environment and for society, which synergizes conservation efforts. However, to date there has been no focused and systematic review of the application of NbS in drylands, despite their importance.

**Fig. 1:**
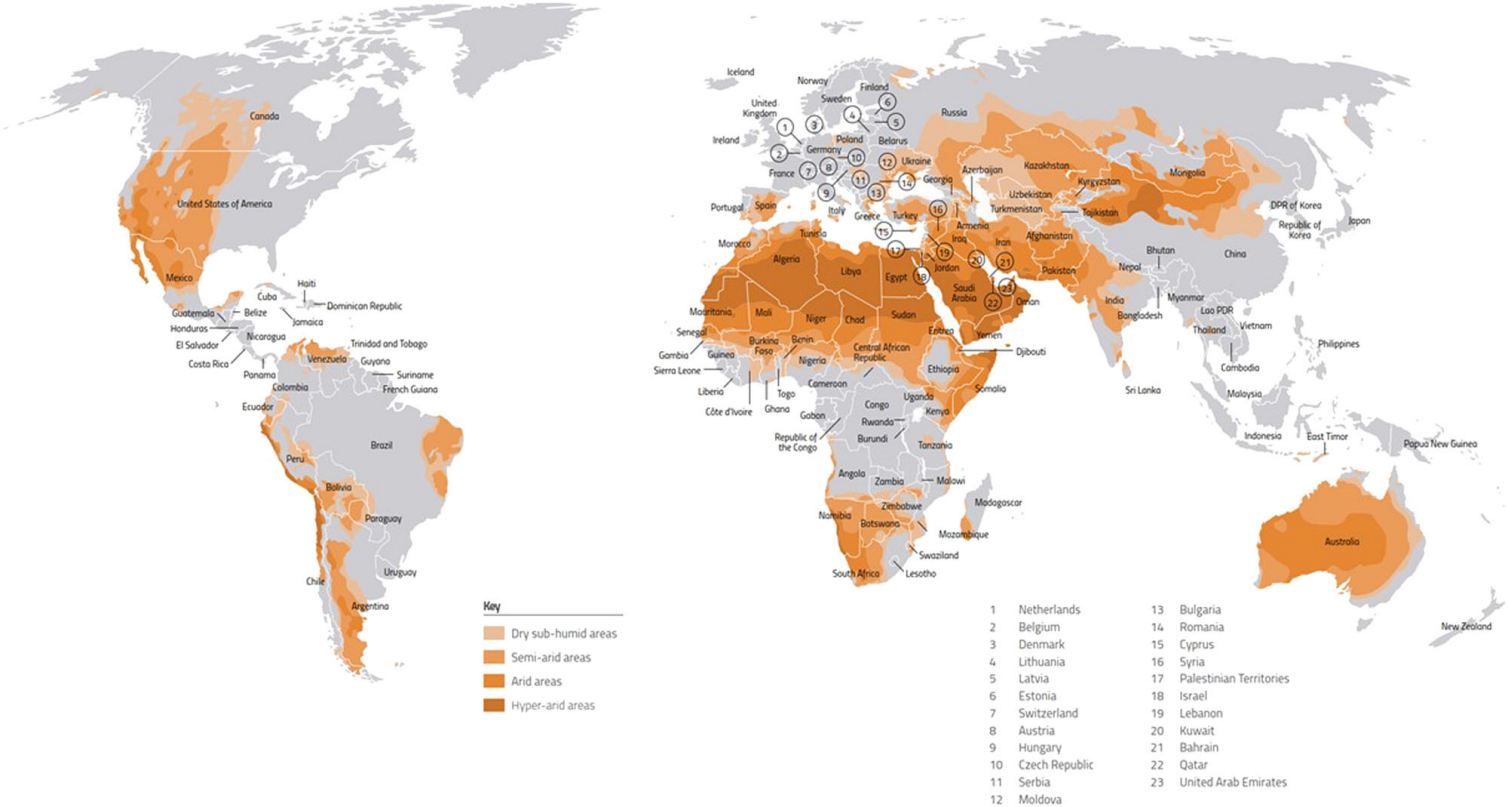
World’s dryland areas (UNCCD, 2017)

Here, we systematically review the application of nature-based solutions in rural drylands around the world with all four types of aridity indices defined by the United Nations: dry sub-humid, semi-arid, arid and hyper arid areas (see Fig. 1 for the world map of drylands). An additional, specific focus of this review is to gather evidence for the feasibility of implementation of NbS and their potential in the Aral Sea region of Uzbekistan, which is an exemplary dryland ecosystem.

### Nature-based solutions, and their relevance to dryland ecosystems

The term nature-based solutions (NbS) reportedly emerged in the late 2000s in connection with the CBD’s “ecosystem approach” method (Cohen-Shacham, et al., 2019), (Agol, Reid, Crick, & Wendo, 2021). Later, the IUCN and the European Commission reframed NbS with the IUCN’s definition being most cited (Cohen-Shacham, et al., 2019), and formally acknowledged recently by the UN during the fifth session of the United Nations Environment Assembly by adopting a *Resolution on nature-based solutions for supporting sustainable development (*UNEP, 2022*)*.

The formal conceptualization defines NbS as “*actions to protect, sustainably manage, and restore natural or modified ecosystems, that address societal challenges effectively and adaptively, simultaneously providing human well-being and biodiversity benefits”* (IUCN, 2016).

**Fig. 2:**
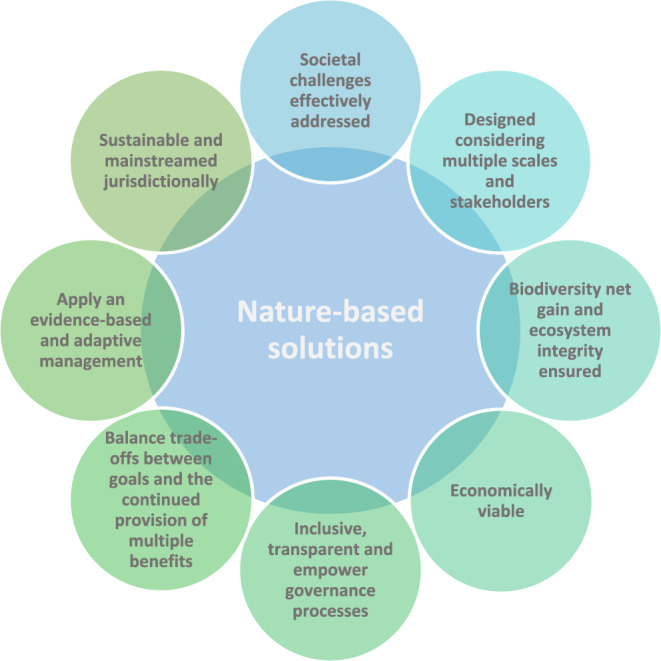
Core criteria of NbS. Adapted from (IUCN, 2020)

IUCN has set out a Global Standard for Nature-Based Solutions (IUCN, 2020), which defines eight criteria for addressing societal, economic, and environmental dimensions for NbS design and implementation (Fig. 2). The Standard stipulates that for an approach to be considered as NbS “*it is imperative for it to provide simultaneous benefits to biodiversity and human well-being*” (IUCN, 2020). To distinguish NbS from other conservation actions, IUCN specifies seven societal challenges, at least one of which needs to be addressed. NbS can often incorporate traditional knowledge, and should ideally involve a wide range of stakeholders - in particular, local communities. Hence, nature-based solutions can be more broadly categorized as managed interventions to restore the environment or ecosystems specifically, by eventually having a positive impact on the local communities.

The relative cost-efficiency of NbS (Bonn, Allott, Evans, Joosten, & Stoneman, 2016), (Girardin, et al., 2021) has led to their increased popularity recently. NbS offer quite a range of benefits (Seddon, et al., 2020), (Girardin, et al., 2021), including biodiversity conservation, ecosystem restoration, climate change mitigation and adaptation. As drylands around the world are projected to become more vulnerable under increasing temperature changes, and Central Asia is expected to be a climate change hotspot in the coming years with increased average temperature and decreased precipitation (Li, Ma, & Zhang, 2020), there is an acute need to mitigate and to adapt to the consequences of climate change by applying sustainable and resilient solutions for which NbS qualify. And NbS is a sustainable approach with minimal negative impacts on the society or the environment (Dubey, Singh, Chaurasia, Pandey, & Singh, 2021). Also, NbS are considered amongst those tools with the greatest potential to reach the UN Sustainable Development Goals (Dubey, Singh, Chaurasia, Pandey, & Singh, 2021), (Gómez Martín, Giordano, Pagano, van der Keur, & Máñez Costa, 2020), which Uzbekistan has committed to achieving by 2030 (The State Committee of the Republic of Uzbekistan on Statistics, 2022).

### The need for nature-based solutions in the Aral Sea region

**Fig. 3:**
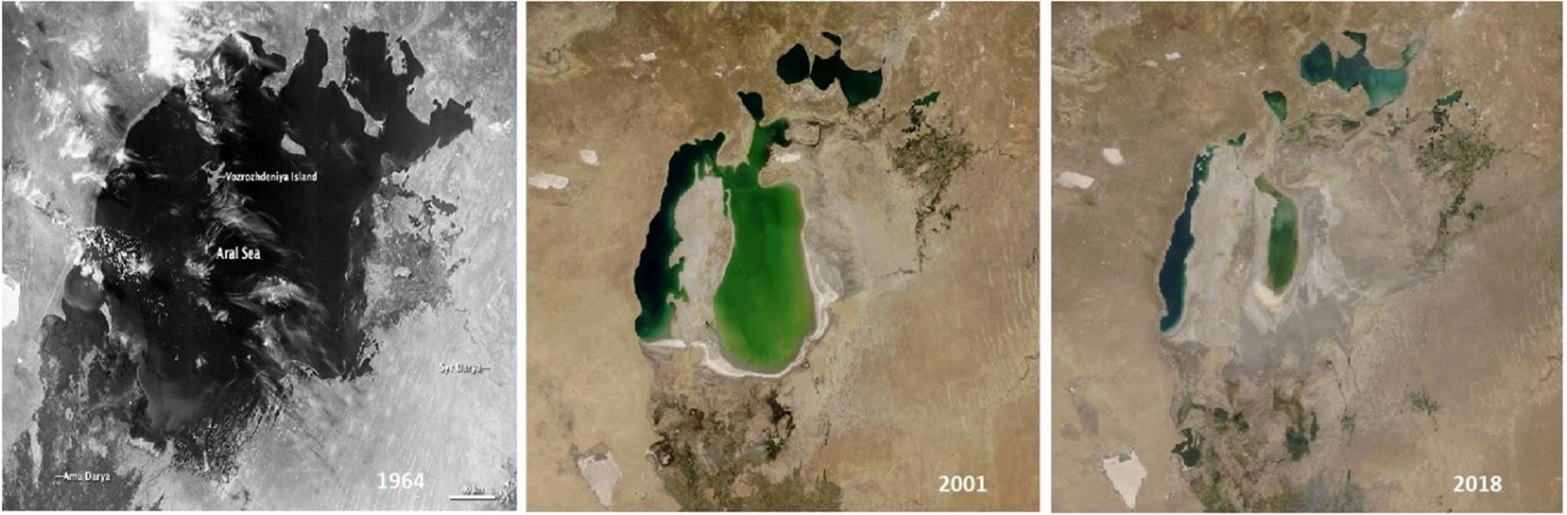
Aral Sea surface area change from 1964 through 2018. Photo credit: NASA Earth Observatory, Google Earth

The Aral Sea—formerly the fourth largest inland lake in the world, and the centre of a thriving fishing industry—has shrunk drastically since the early 1960s due to the misuse of river flow for irrigation, and is now represented by a highly saline southern Aral Sea in Uzbekistan and northern Aral Sea in Kazakhstan. The sandy desert in the very east of Uzbekistan, covering a territory of about 5,5 million hectares (Summary report on the LDN target setting programme in the Republic of Uzbekistan, 2019), the former Aral Sea left behind, is now widely referred to as the Aralkum desert (Fig. 3).

The study area, the Uzbek part of the Aral Sea region, is characterised as a dryland by the UNCCD (UNCCD, 2017), and, specifically, a dry sub-humid area. Both the ecosystem and the human population of the region are under considerable pressure due to the consequences of the Aral Sea desiccation. Along with a newly formed desert ecosystem, the desiccation has brought about a number of environmental, social and economic challenges to the region, impacting millions of people residing in the Aral Sea basin (Lemly, Kingsford, & Thompson, 2000). The far-reaching negative consequences of the Aral catastrophe include, but are not limited to: increased number of lung, kidney and thyroid diseases, infant mortality and morbidity, decreased life expectancy, frequent dust storms, loss of aquatic and terrestrial biodiversity, diminished ecosystems (i.e., riparian forests, wetlands, grasslands) and their services, massive job losses due to the collapse of the fishing and tourism industries (Lemly, Kingsford, & Thompson, 2000), (Li, Ma, & Zhang, 2020), (Lioubimtseva & Henebry, 2009), (Schlüter, et al., 2013). The government of Uzbekistan has officially stated that it will now be impossible to restore the Aral Sea to its former (pre-1960) state, and has instead committed to mitigate the consequences of this ecological disaster on human wellbeing, environment and the local economy. Given the multitude of environmental challenges the Aral Sea region countries are facing, sustainable and cost-efficient approaches are needed to secure the well-being of the local population and environmental resilience.

## Method: Literature Review

We conducted a systematic review on the topic of nature-based solutions in terrestrial dryland ecosystems around the world, with a specific focus on the Aral Sea region in Uzbekistan. ‘Wetlands’ were included as a search term to ensure we captured those that are nested into larger dryland landscapes, including the following inland water bodies: lakes, ponds, and ephemeral wetlands. Further, wetlands nested within larger dryland landscapes are reported to be among the ecosystems most effected by climate change (Parra, et al., 2021), (Williams, 1999), hence are in need of urgent conservation action.

The focal case study area—the Aral Sea—primarily transitioned from one of the largest inland water bodies in the world into a sandy desert over the course of several decades, still contains the small remnant ‘southern Aral Sea’, the existence of which remains under threat due to the absence of water inflow and high evaporation rates. Also, wetlands in drylands are considered crucial for regional biodiversity (Williams, 1999), and the vital importance of wetlands and ecosystem services they provide in dry areas such as the Aral Sea region cannot be neglected. This is both for biodiversity conservation, and also ecosystem services, due to the dependence of the local human population on water resources and interconnectedness of the state of terrestrial ecosystems (e.g., rangelands/grasslands, afforested areas) with the availability of local water bodies and water resources as such.

### Review of NbS Approaches in Dryland Ecosystems

A multilingual thematic keyword search was conducted in January – March 2022 using Boolean operators with the following word combinations (TITLE-ABS-KEY) AND (LIMIT-TO (LANGUAGE, “English”, “Russian”)): “nature-based solutions AND terrestrial”, “ecosystem-based and drylands”, “nature-based solutions AND dryland”, “nature-based solutions AND heathland”, “nature-based solutions AND desert”, “nature-based solutions AND rangeland”, “nature-based solutions AND grassland”, “nature-based solutions AND wetland”, “nature-based solutions AND protected area”. Information was sourced from two major scientific publication databases—Scopus, and Web of Science—to ensure the most comprehensive capture of articles possible. The results from both publication databases were downloaded in a BibTex format, sorted into eighteen subject-specific databases corresponding to keywords used in JabRef reference manager software, then merged to cross-check for duplicates, and further content analysis was carried out.

Results on blue and/or green urban infrastructure, coastal ecosystems, and on areas not considered as drylands by the UNCCCD (2017) were excluded. All sources of publications were considered in the review. Search results included English and Russian languages for both search engines, as a majority of the research performed historically in Central Asia has been reported in the Russian language; results returned in any other languages were excluded (due to linguistic limitations of the research team). Since there is no universal definition or translation of the term “nature-based solutions” in Russian, no separate keyword search was conducted in Russian language.

To complement the systematic review, a non-systematic review was conducted into widely adopted NbS definitions and criteria for the selected ecosystem types. The language used for the non-systematic literature review was English only (as the Russian language does not distinguish and define NbS specifically). The non-systematic review primarily focused on the grey literature (i.e., the IUCN handbooks and guidance, as well as the UN resolution papers).

The final set of publications was analysed thematically as they were grouped into dryland ecosystem types (i.e., rangelands/grasslands, heathland, desert, wetlands) according to the search keywords used. Publications that did not include “nature-based solutions” as a specific term, but included the term “ecosystem-based approaches” were still considered based on the content and relevance to the subject area and the geographic focus.

### Review of NbS Approaches in the Aral Sea Region

An additional nested search was performed to expand the general results to the geographic case study region of focus, using the following combinations (TITLE-ABS-KEY) AND (LIMIT-TO (LANGUAGE, “English”, “Russian”)): “nature-based AND solutions AND Central Asia”, “nature-based AND solutions AND Aral Sea”, “ecosystem-based AND Aral Sea”, “rangeland AND Aral Sea”, “grassland AND Aral Sea”, “wetland AND Aral Sea”. Additional keywords were used to include the most frequent interventions in the Aral Sea region mentioned in the literature: “afforestation AND Aral Sea”, “saxaul AND Aral Sea”, “artemia AND Aral Sea”. As in the case above, the search results covered both English and Russian languages.

## Results

The systematic search on NbS implementation in dryland ecosystems resulted in 2557 non-duplicative scientific publications, 2451 of which were filtered out as being irrelevant (see Appendix 1 for detailed information). A final set of 106 papers were retained and read in their entirety.

Content analysis suggests that the NbS approach has been poorly documented in dryland ecosystems. The largest number of studies on NbS application originated from China. There were a few publications that mentioned the Aral Sea basin and Central Asia in the literature without country-specific information or a case study.

The additional nested search on the focal case study area, the Aral Sea region of Uzbekistan, resulted in 151 non-duplicative records, with 40 being considered as relevant (Appendix 2).

Types and benefits of nature-based solutions identified—both for dryland ecosystems in general, and for the Aral Sea region specifically—were compiled into Table 1 based on the functions they perform in improving ecosystem service provision, and/or providing benefits for humans and increasing well-being. The final list was formed based on the recently formalized definition of nature-based solutions by the IUCN neglecting small-scale interventions, which in some cases the authors considered to represent NbS.

**Table 1:** NbS discussed in terms of application to dryland ecosystems

Types of nature-based solutions relevant to drylands	Potential benefits	Source
Afforestation/reforestation using natural vegetation	• Carbon capture.	(Seddon, et al., 2020), (Mills, et al., 2020), (Zhang, Sun, Huettmann, & Liu, 2022), (Yao, Fu, Liu, Wang, & Song, 2021), (Chausson, et al., 2020), (Di Sacco, et al., 2021), (Djoudi, Brockhaus, & Locatelli, 2013), (Schachtsiek, Lamers, & Khamzina, 2014).
	• Biodiversity conservation.	
	• Microclimate stabilization.	
	• Wind and soil erosion prevention.	
	• Sand fixation.	
	• Source for fuelwood.	
	• Soil salinity reduction.	
	• Soil erosion reduction.	
	• Natural vegetation succession in the vicinity thanks to soil nutrient improvement.	
	• Climate change adaptation.	
	• Landslide reduction.	
	• Emissions mitigation.	
	• Alternative livelihood opportunities.	
Agroforestry (with reference to irrigated areas/croplands)	• Soil erosion reduction.	Adapted from (Seddon, et al., 2020), (Di Sacco, et al., 2021), (Plieninger, Muñoz-Rojas, Buck, & Scherr, 2020) (Aleksandrova, Lamers, Martius, & Tischbein, 2014), (Lamers, Bobojonov, Khamzina, & Franz, 2008), (Toderich, et al., 2013b), (Kumar, Khamzina, Knöfel, Lamers, & Tischbein, 2021).
	• Soil fertility improvement.	
	• Adaptation to climate change.	
	• Higher economic returns through income diversification.	
	• Energy security enhanced (fuel wood, timber).	
	• Reducing exposure to heat and drought.	
	• Food security (fruit) enhanced.	
	• Groundwater level stabilization.	
	• Biodiversity restoration.	
	• Local livelihoods improvement.	
Restoration of wetlands/peatlands	• Water supply regulation.	Adapted from (Seddon, et al., 2020), (Belle, Collins, & Jordaan, 2018), (Thorslund, et al., 2017), (Taillardat, Thompson, Garneau, Trottier, & Friess, 2020), (Bekele & Haile, 2021), (Tanneberger, et al., 2021).
	• Reduced exposure to soil erosion.	
	• Reduced number of landslides.	
	• Reduced flood risk.	
	• Biodiversity protection through habitat rehabilitation.	
	• Food security improvement (e.g., fish catch, reed harvesting for forage).	
	• Support of the local livelihoods.	
	• Climate change mitigation and adaptation.	
	• Drought mitigation.	
	• Carbon sequestration.	
	• Groundwater level regulation.	
	• Coastal protection.	
	• Soil moisture regulation.	
	• Human health support.	
	• Provision of cultural services, recreation, and tourism opportunities.	
	• Wildlife habitat improvement and biodiversity conservation.	
	• Livelihood improvement and additional job opportunities.	
	• Sustainable food and fodder production.	
	• Improved provision of ecosystem services.	
Rewilding	• Biodiversity conservation.	(Sweeney, et al., 2019)
	• Restoring ecosystem processes.	
Grassland/pastureland/range-land restoration	• Increased climate resilience.	(Yao, Fu, Liu, Wang, & Song, 2021), (Wang, Yan, Xue, Batunacun, & Liu, 2022), (Toderich, et al., 2013b).
	• Food security enhanced.	
	• Provision of economic benefits.	
	• Vegetation sand control.	
	• Mitigation of desertification.	
Protected area establishment	• Biodiversity conservation.	Adapted from (Carroll & Ray, 2021), (Cohen-Shacham, et al., 2019), (MacKinnon, Dudley, & Sandwith, 2011)б (Oberle, Mackinnon, & Sandwith, 2021)
	• Climate change mitigation and adaptation (e.g., carbon storage).	
	• Ecosystem services enhancement (e.g., water supply).	
	• Landscape restoration.	
	• Habitat connectivity.	
	• Protecting ecosystem resilience.	
	• Reducing risks of and impacts. from extreme events.	
	• Places of recreation.	

### Nature-Based Solutions Relevant to Dryland Ecosystems

**Fig. 4:**
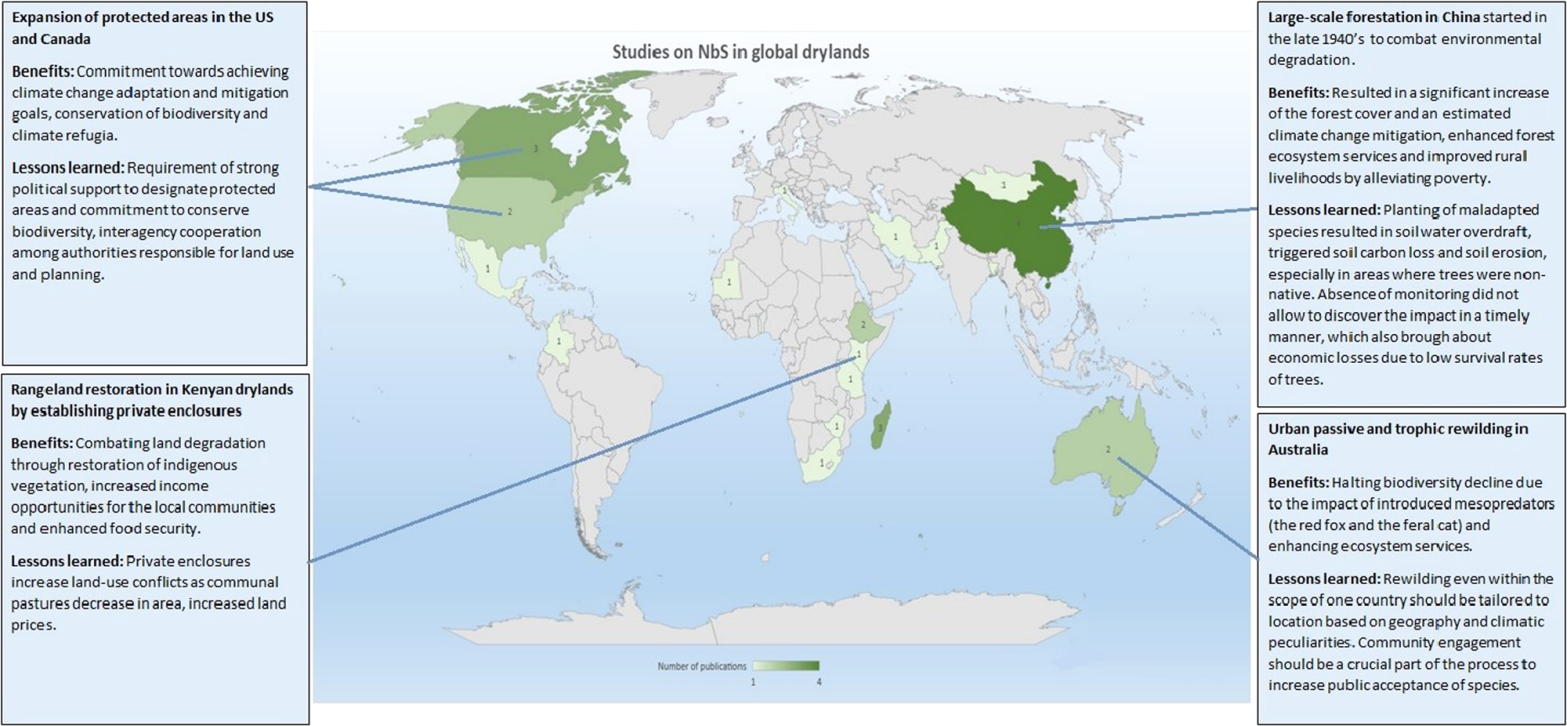
Number of studies covering NbS application in global drylands with selected examples

Our review of nature-based solutions in rural dryland ecosystems returned a limited number of case studies of actual NbS application; particularly those that described specific outcomes and lessons learned, demonstrated success and socio-economic benefits, and sustained results. Many cases reported on not the actual implementation and outcomes of NbS, but rather on the potential of adopting specific approaches (e.g., agroforestry/intercropping on marginal lands, application of halophytes to restore degraded soil on grazelands) as a tool to rehabilitate degraded land and wetlands. The most common cases of ecosystem-based approaches (Cohen-Shacham, et al., 2019), an alternative to NbS suggested widely in the literature reviewed, appeared to be afforestation, agroforestry and wetland restoration with the prevailing number of studies originating from China (Fig. 4).

Afforestation measures are discussed as a cost-effective nature-based solution for combatting desertification, adapting to and mitigating climate change impacts through increased carbon sequestration potential (Seddon, et al., 2020) and halting biodiversity loss, as well as improving livelihood opportunities of the local communities (Zhang, Sun, Huettmann, & Liu, 2022), (Di Sacco, et al., 2021), (Djoudi, Brockhaus, & Locatelli, 2013). However, authors (Zhang, Sun, Huettmann, & Liu, 2022) argue that afforestation activities can also fail both economically and environmentally as it was in the case of China and the planting of maladapted species, along with an absence of post-forestation monitoring to check on the survivability of trees. Failure among afforestation efforts was more frequent compared to reforestation due to the fact that trees did not naturally occur in areas where they were planted. Also, plantations of non-native monocultures were reported to be of low value for the revival of the local biodiversity (Zhang, Sun, Huettmann, & Liu, 2022), and demonstrated increased chances of susceptibility to diseases and pests (Seddon, et al., 2020). Due to the impact of afforestation on soil carbon both positively and negatively (Seddon, et al., 2020), selection of appropriate sites and species turn out to be a key to success, but most importantly, afforestation should be implemented with the application of native and/or endemic species to avoid introduction of invasive alien species.

Agroforestry is mostly proposed to rehabilitate marginal and salt-affected irrigated lands. Agroforestry can significantly contribute to enhancing food security in where there is water scarcity and climate change impact in drylands, diversifying income opportunities, restoring biodiversity, enhancing carbon sequestration and reducing pressure on natural forests where they occur (Di Sacco, et al., 2021), (Aleksandrova, Lamers, Martius, & Tischbein, 2014), (Lamers, Bobojonov, Khamzina, & Franz, 2008). However, it is notable that agroforestry in croplands has been erroneously proposed as an afforestation measure in some papers reviewed.

Rangeland restoration is largely discussed as a climate mitigation option due to the carbon storage potential of grasslands (Yao, Fu, Liu, Wang, & Song, 2021), as well as a means of improved livelihood opportunities for the local pastoralists in degraded areas and a measure to combat desertification and land degradation (Toderich, et al., 2013a). Examples of grassland restoration activities given as NbS options, i.e., prohibition of grazing in some areas of China (Yao, Fu, Liu, Wang, & Song, 2021), were reported to have compromised herders’ income opportunities. Hence, these examples do not fully satisfy NbS definition framed by the IUCN, which stipulates provision of societal benefits as a result of NbS approaches.

Wetland restoration is a promising tool in many areas that could potentially compete with afforestation/reforestation in terms of carbon sequestration and climate change mitigation potential (Taillardat, Thompson, Garneau, Trottier, & Friess, 2020), (Seddon, et al., 2020), (Tanneberger, et al., 2021). Besides, wetlands perform multiple other ecosystem services such as disaster risk reduction (e.g., they can function as fire breaks), enhancement of food security, and biodiversity conservation (Belle, Collins, & Jordaan, 2018). Wetlands in some arid areas depend on the river runoff and inflow, which is used for agricultural purposes and by decreasing the areas under some water-intensive crops (e.g., cotton and rice) (Schlüter, et al., 2013), wetlands are likely to receive inflow to keep their state stable. Overall, the literature reviewed suggests an enormous potential of wetland restoration with multiple benefits summarized in Table 1. However, (Taillardat, Thompson, Garneau, Trottier, & Friess, 2020) argue that conserving existing wetlands is more cost-efficient as opposed to their restoration in terms of contribution to climate change mitigation, large initial investments, and subsequent operation costs.

Rewilding as an option to restore local biodiversity can have promising outcomes in eliminating damages caused by introduced species. Sweeney et al. (2019) suggest that rewilding, due to its purported environmental and societal benefits, “shares similarities with nature-based solutions”. We consequently include it as a type of nature-based solution in this review. Rewilding should, however, be locally tailored not only within the context of a region or a continent, but also within the context of one single country (Sweeney, et al., 2019), as the number of factors influencing rewilding success may differ spatially.

Protected areas can perform numerous benefits by preserving ecosystems and the services flowing from them, provided they are managed effectively (Oberle, Mackinnon, & Sandwith, 2021), (Carroll & Ray, 2021), (MacKinnon, Dudley, & Sandwith, 2011). In some cases, it has been demonstrated that protected areas can, among other things, provide human health benefits and thus can offset healthcare costs (Oberle, Mackinnon, & Sandwith, 2021). Again, for this reason we include them as an NbS on this review. However, it is worth underlining that the effectiveness of protected/conserved areas cannot be equated to their existence only, management is a key to ensuring both environmental and societal benefits are gained to qualify to be considered as a NbS.

Summarizing lessons learned from the literature sources discussed above, we conclude that proper management and monitoring is required even for small scale activities to ensure their efficiency and acceptance by communities that have been benefiting from the use of natural resources and which they treat as a communal resource. Thorough socio-economic analysis has proved to be critical for most NbS activities before the start of any project to minimize community resistance to planned interventions (Mills, et al., 2020). Adaptive management, community awareness raising on functions of ecosystems and natural resources in general and their involvement in the planning and implementation processes are highly likely to increase the success of NbS interventions (Belle, Collins, & Jordaan, 2018). Success of EBA/NbS projects is quite difficult to evaluate, which is why it is essential to document every evidence (Mills, et al., 2020).

### Nature-based Solutions Relevant to the Aral Sea region

Based on the focussed case study literature review we conducted, we now focus on discussing four of the above-mentioned major nature-based solutions, which are relevant to the Uzbek part of the Aral Sea region. These are afforestation, wetland restoration, rangeland restoration and protected areas establishment in Karakalpakstan, an autonomous region in the far-western part of Uzbekistan.

#### Afforestation using native tree species

The governments of Uzbekistan and Kazakhstan have been undertaking large-scale afforestation activities on the dried-out Aral Seabed over the past several decades^1^ to mitigate the impacts of frequent dust storms both on the population and the environment. There is a whole state program for the development of the Aral Sea region in Uzbekistan as a zone of ecological innovations and technologies supported by a special Resolution of the United Nations’ General Assembly (United Nations General Assembly, 2021), which stipulates an increase in the area of afforested lands in the former seabed. As large-scale afforestation of the former Aral seabed is planned to continue through 2030, as stipulated by the Decree of the Cabinet of Ministers of the Republic of Uzbekistan^2^, there is at the very least a need for planning and monitoring, as well as selection of native species to ensure afforestation success.

Shrublands/afforested areas, to which the sandy seabed is planned to be converted to, are reported to maximize sand fixation and minimize erosion (Löw, et al., 2021), (Kim, et al., 2020). The most prominent native species in terms of the ability to grow in sandy, loamy and saline soils are reported to be Saxaul (*Haloxylon*), *Salsola Richteri* (Moq) Karel ex Litv and *Calligonum caput-medusae* Schrenk (Bakirov, Khamzaev, & Novitskiy, 2020), *Krascheninnikovia eversmanniana* and *Artemisia ferganensis (*Shomurodov, et al., 2021*)*. Upon reaching certain heights and within a few years, these species are able to reduce wind erosion and subsequent dust storms drastically. Also, topsoil quality was reported to have improved in saxaul plantations, which bonds sand particles together with the help of debris (Bakirov, Khamzaev, & Novitskiy, 2020). In addition, plants used for afforestation in the northern Aralkum in Kazakhstan were documented to have improved soil quality by reducing the soil salinity level and enhancing soil enzyme activities, however, monitoring might be needed for at least two decades to detect trends (An, Chang, Han, Khamzina, & Son, 2020). However, within the dried-out Aral Seabed, there are different types of soils, some of which are not suitable for afforestation due to the high salt and sand contents (Bakirov, Khamzaev, & Novitskiy, 2020), (Shomurodov, et al., 2021). It has also been documented that sub-soil conditions are at times more important than topsoil for tree growth (Matsui, Watanabe, Kussainova, & Funakawa, 2019). Hence, a number of indicators, such as soil salinity and soil moisture, and terrain need to be considered while planning afforestation (Löw, et al., 2021), (Kim, et al., 2020).

Afforestation of irrigated cropland in combination with agroforestry in the Aral Sea basin is recommended by a number of authors (Djalilov, Khamzina, Hornidge, & Lamers, 2016), (Dubovyk, Menz, & Khamzina, 2016), (Khamzina, Lamers, Worbes, Botman, & Vlek, 2006), (Kumar, Khamzina, Knöfel, Lamers, & Tischbein, 2021) as a means to reduce soil salinity on degraded agricultural lands, which could be another solution to reduce salinity and improve soil quality of abandoned croplands.

There is scant evidence of research and development activities happening before or after afforestation in the Aralkum desert in the literature, beyond the publications cited above. Monitoring of afforested lands and soil quality monitoring could have substantially improved survival rates of saxaul and other shrubby plantations in the Aralkum. Large-scale afforestation as NbS in the dried-out Aral Sea basin presents opportunities for further research in lieu of absence of documented evidence on the environment, both positive (e.g., the extent of sand transfer reduction) and negative (e.g., impact on groundwater resources).

#### Wetland restoration

Wetlands, as a potentially cost-effective nature-based solution (Seifollahi-Aghmiuni, Nockrach, & Kalantari, 2019), provide a range of ecosystem services, including food security, climate adaptation and mitigation by acting as natural carbon sinks, and biodiversity conservation (Thorslund, et al., 2017), (Seifollahi-Aghmiuni, Nockrach, & Kalantari, 2019), (Taillardat, Thompson, Garneau, Trottier, & Friess, 2020). Being subjected to extensive degradation recently, wetlands in the Aral Sea basin, just like in most parts of the world, are being continuously lost (Thorslund, et al., 2017). Restoration of wetlands in the Aral Sea basin would not just have an economic value for the local population, but also environmental benefits (Girardin, et al., 2021), and would substantially contribute to achieving the UN Sustainable Development Goals (Seifollahi-Aghmiuni, Nockrach, & Kalantari, 2019).

Uzbekistan currently has three wetlands included in the list of Wetlands of international importance (The Ramsar Convention Seceretariat. Annotated List of Wetlands of International Importance: Uzbekistan, 2022), however, there are none located in Karakalpakstan. Nonetheless, wetlands in the Aral Sea basin serve as an important habitat both for inland waterbirds and for the migratory species in Central Asia (Kasprzykowski, Goławski, Mitrus, & Stański, 2014). Besides performing ecological functions, local wetlands are vital sources of income for the population by supporting fishing, hunting, livestock husbandry through the provision of forage (Lemly, Kingsford, & Thompson, 2000), (Schlüter, et al., 2013).

Wetlands in the Aral Sea basin were reported to have been impacted by anthropogenic activities that led to the degradation of vegetation in the riparian zone (Jiang, Jiapaer, Bao, Guo, & Ndayisaba, 2017), (Kasprzykowski, Goławski, Mitrus, & Stański, 2014). Aggravating climate change (Ragab & Prudhomme, 2002) and unsustainable agricultural practices, as well as desiccation of the Aral Sea are reported to have had further negative consequences on the wetlands in the Amu Darya and Syr Darya river basins, and thus on the avifauna of the wetlands in the region (Kasprzykowski, Goławski, Mitrus, & Stański, 2014), (Lemly, Kingsford, & Thompson, 2000), (Schlüter, et al., 2013).

Wetlands in the Aral Sea basin are reported to be unstable in terms of their hydrological regimes and are highly dependent on the water inflow, which in turn defines their salinity levels and ability to support native biodiversity (Schlüter, et al., 2013). During dry years these wetlands tend to almost dry out (Schlüter, et al., 2013). As reported by Schlüter, et al. (2013), fluctuations in the water table of wetlands can kill roe that fish lay on the shores with the already shallow water table. This in turn defines fish abundance and eventually impacts local biodiversity and livelihoods.

The only species that currently survives in what is known as the southern Aral Sea in Uzbekistan is brine shrimp (*Artemia*). Artemia, which is used as forage for aquatic species, is currently being harvested and represents business opportunities for the local population (Aladin, et al., 2018), (Arashkevich, Sapozhnikov, Soloviov, Kudyshkin, & Zavialov, 2009). Survival and reproduction of Artemia are influenced by a number of factors, among which the most significant is the salinity and water temperature (Marden, et al., 2012), (Qi, et al., 2021), (Aladin, et al., 2018). The government of Uzbekistan has been issuing quotas for artemia harvesting in the Aral Sea (Marden, et al., 2012), but in 2022 the quota was substantially reduced, likely because the artemia populations are also decreasing (Anonymous, 2022).

Since brine shrimp in the wetlands of the Aral Sea basin attract birds to feed, including flamingo (Aladin, et al., 2018), (Qi, et al., 2021), and the fact that artemia cultivation and harvesting provides local livelihood opportunities, suggest that it could provide a basis for some form of nature-based solution. The issues with human-biodiversity interaction in wetlands, where artemia is grown and harvested, sustainability of yields, water availability and its quality yet need to be researched to comply with NbS criteria.

The Agency of the International Fund for Saving the Aral Sea, established in 1998 specifically to implement projects of the Aral Sea Basin Programs (Agency of the International Fund for Saving the Aral Sea, 2022a), has been undertaking activities on restoring and creating wetlands in the southern Aral Sea region with the financial support of the government of Uzbekistan since 1995 (Agency of the International Fund for Saving the Aral Sea, 2022b). However, according to the list of activities implemented to restore wetlands, i.e., construction and reconstruction of dams, channels, and outlets, the efforts have been focused on engineering activities. Also, considering the ever-ongoing shortage of water resources and degradation of wetlands in the Aral Sea basin, there is a question of sustainability and cost-efficiency of the results achieved for almost three decades now. As reported by Li et al. (Li, Chen, Zhang, & Pan, 2019), there is an ongoing drastic shrinkage of water bodies in Karakalpakstan leading to the loss of invaluable ecosystem services they provide.

#### Rangeland restoration

The recent study by the UN (ILRI, 2021) reveals that rangelands occupy 54% of the global terrestrial surface and cover about 57% of the territory of Uzbekistan (Toderich, et al., 2013a). Rangeland/grassland restoration can reduce net soil CO2 emissions, conserve biodiversity, soil and water resources (Abdalla, Mutema, Chivenge, Everson, & Chaplot, 2022), (Li, Chen, Zhang, & Pan, 2019), and thus have a climate mitigating factor. The health of grasslands all over Central Asia has been severely compromised by anthropogenic activities and climate change over the past several decades (Li, Chen, Zhang, & Pan, 2019). According to Li et al. (Li, Chen, Zhang, & Pan, 2019), the decline of ecosystem service value in Karakalpakstan (including those of grasslands) was the highest among all researched areas in Central Asia; a trend which seems likely to continue.

Desertification of rangelands in the study area has been ongoing due to the misuse of resources (mainly overgrazing), oil and gas exploration, climatic factors (reduced precipitation and droughts), overharvesting of fuel wood, unsustainable agricultural activities (Jiang, Jiapaer, Bao, Guo, & Ndayisaba, 2017), (Toderich, et al., 2013a), (Shaumarov, et al., 2012). All these have led to increased levels of soil and water salinity, as well as to the reduction of the water table of desert pastures in the Aral Sea basin (Toderich, et al., 2013b). Rangelands in the Aral Sea basin represent not just significant economic and environmental value to local stakeholders, but also cultural values (Shaumarov, et al., 2012). Hence, the degradation of rangelands in Central Asia represents a substantial threat to the communities who rely upon them, having been long engaged in pastoralism.

Toderich et al. (Toderich, et al., 2013a) suggest that the best way to rehabilitate saline soils in desert rangelands is to plant salt-resistant wild halophytes, which represent a low-cost solution. Afforestation using native salt- and drought-tolerant species is another alternative to restore degraded desert landscapes, which has direct applicability to the dried-out Aral Seabed (Bakirov, Khamzaev, & Novitskiy, 2020), (Toderich, et al., 2013b), (Shomurodov, et al., 2021). In the long term, rehabilitation of degraded rangelands in the Aral Sea basin has a potential to improve livelihood options of local pastoralists.

The review has not returned specific rangeland rehabilitation methods or success stories in the Aral Sea basin. The potential for rangelands to provide a basis for NbS—given that they are one of the major potential carbon sinks in dryland areas such as the Aral Sea basin—requires detailed further research.

#### Protected areas in Karakalpakstan

Protected areas (PA) that lead to biodiversity conservation and climate mitigation benefits could represent a cost-effective nature-based solution (Mackinnon, Mrema, Richardson, Cooper, & Gidda, 2021), (Roberts, O’Leary, & Hawkins, 2020). As the number of ecological issues associated with biodiversity loss, climate change, food security is expected to grow in the coming decades, protected areas are seen as an effective adaptation and mitigation tool (MacKinnon, Dudley, & Sandwith, 2011).

PAs can perform multiple functions and encompass several NbS approaches with respect to the ecosystem services they represent (see Table 1 for a list of services provided by PAs). As part of the Aichi Biodiversity Target 11, to which Uzbekistan has committed (CBD-UNDP, 2021), protected areas highly contribute to achieving the UN Sustainable Development Goals. To reach its commitments to increase the percentage of protected areas with respect to the total territory, the government of Uzbekistan almost tripled the area of PAs in Karakalpakstan in 2021–2022 alone. Currently, there are five protected areas in Karakalpakstan as listed below in the order of establishment:

- ***Lower Amu Darya Biosphere Reserve***^3^– total area: 687.18 km^2^, year of designation: 2011.
- ***Saigachiy complex (landscape) nature reserve***^4^– total area; 6283 km^2^, year of designation: 2016.
- ***South Ustyurt National Park***^5^– total area: 14471.43 km^2^, year of designation: 2020.
- ***Sudochye-Akpetki state wildlife sanctuary***^6^– total area: 2805.07 km^2^, year of designation: 2021.
- ***Aralkum national park***^7^– total area: 10000 km^2^, year of designation: 2022.

We were unable to locate any published management plans associated with the protected areas in Karakalpakstan, and as the effectiveness of a protected area very much depends on how it is managed (MacKinnon, Dudley, & Sandwith, 2011), (Roberts, O’Leary, & Hawkins, 2020), it is consequently unclear how these five existing protected areas will fulfil their tasks.

## Discussion: Gaps and Opportunities

The application of nature-based solutions, such as the large-scale afforestation of the Aral Seabed, potentially creates alternative income sources for the local population. For instance, local residents in Muynak region of Karakalpakstan get hired by the State Forestry Committee to collect the seeds of saxaul plants on a seasonal basis. However, large-scale afforestation in deserts/arid areas without proper planning can lead to massive economic losses and to very low plant survival rates, as some cases around the world (Zhang, Sun, Huettmann, & Liu, 2022) demonstrate. The issue with low plant survival rates on the dried-out seabed in Uzbekistan is not uncommon: according to Shomurodov et al., (2021), survival rate of plant species in afforested areas is as low as 20%. As a result, the same areas of land are sown several times over a number of years to increase the vegetation cover. This may not be a promising investment in terms of time, human and financial resources unless timely post-afforestation monitoring and R&D on the quality of land is ensured. Lack of monitoring is another issue determining the success of afforestation in the Aral Sea region. The International Innovation Centre for the Aral Sea Basin, which among others is carrying out testing and planting trees in the Aral Seabed, confirmed they do not have enough human capacity and financial resources to undertake monitoring of afforested areas (Anonymous, 2022), although they had announced a country-wide fundraising campaign to plant trees and had received monetary donations both from individuals and organizations 8^,^^9^.

The water consumption associated with afforestation is another issue that needs to be further evaluated (Tew, Vanguelova, & Sutherland, 2021), (Yao, Fu, Liu, Wang, & Song, 2021), (An, Chang, Han, Khamzina, & Son, 2020), as there is a high threat of depleting groundwater resources in dry areas through afforestation (Kumar, Khamzina, Knöfel, Lamers, & Tischbein, 2021), (Zhang, Sun, Huettmann, & Liu, 2022). Hence, water availability needs to be considered thoroughly when it comes to planning desert greening activities, as there is large-scale evidence of failed or low effective interventions aimed at greening the Aral Seabed. Considering that the main soil types in the exposed seabed are solonchaks (with high concentration of soluble salts) and takyrs (heavy-textured soils under arid conditions)^10^, that have low organic carbon content (An, Chang, Han, Khamzina, & Son, 2020), monitoring of afforested lands is critical to better examine the impact of tree plantations on soils (An, Chang, Han, Khamzina, & Son, 2020). Also, if afforestation is not based on a scientifically sound selection of plant species and is comprised of monocultures only, the efforts may end up being unsustainable in terms of vulnerability to various diseases and a limited carbon capture capacity of an individual plant (Seddon, et al., 2020), as well as limiting potential biodiversity benefits. So, policymakers and scientists must collaborate to ensure that only native plant and tree species are used for afforestation, if the intervention is to count as a NbS.

Wetland restoration in the Aral Sea basin has a number of benefits both for the people and the biodiversity, and yet requires critical evaluation in terms of costs (e.g., water diversion, financial investments, sustainability, self-sustainment and etc.) versus benefits (e.g., fisheries development, biodiversity restoration, income opportunities and etc.). According to Schlüter et al. (Schlüter, et al., 2013), some of the wetlands in the Aral Sea region are being maintained using drainage water. However, the long-term impact of drainage water, most importantly its quality, on the biodiversity of wetlands does not seem to have been thoroughly examined to tell whether the approach can be justified. Moreover, literature review explicitly revealed that wetland restoration in the Aral Sea basin has never been documented as a nature-based solution, nor has rangeland restoration been.

Protected areas, as a means of environmental restoration on a wider landscape scale by encompassing multiple ecosystem types, seem to have more of a quantity rather than a quality approach in Uzbekistan. Existing protected areas need to be explored for the opportunities to be connected through green corridors to ensure better habitat connectivity for migratory animal species.

Overall, in our view, the most common possible issues of NbS application in the Aral Sea basin requiring further in-depth research are the following:

- Resilience of afforested areas to weather changes, temperature fluctuations, water availability and their long-term sustainability.
- Sustainability and cost-effectiveness of wetlands restoration in the Aral Sea region of Uzbekistan.
- Governance challenges in terms of cooperation among various agencies to effectively conduct research and development, and subsequent monitoring of interventions implemented.
- Planning and management of protected areas, their integrity with the landscape, animal migratory routes and areas of anthropogenic activities.
- Availability of actual data on the outcomes of implemented efforts, namely afforestation and wetland rehabilitation to minimize duplication of efforts and to provide a basis for scientific research.

## Conclusion

To our knowledge, this is the first attempt to systematically review nature-based solutions in dryland ecosystems, and certainly the first with a specific geographic focus on the Aral Sea region of Uzbekistan as a case study.

Afforestation, as recently seen as a major viable solution to rehabilitate the ecosystems of the dried-out Aral Seabed, has a number of benefits and yet challenges that need to be balanced out before any decision is made on increasing investments in plantations. In fact, new ecosystems were created twice in the place of once fourth largest inland water body on the planet: a desert (Aralkum) was formed initially, which is now being replaced by afforested areas. So, the ecology of the region has been altered drastically in a very short period of time (several decades). It is clear that deeper research is needed in terms of the impact these ecosystem changes might have in the long term, as well as their self-sustaining capacities, and the timespan it takes to balance out the investments and the potential benefits. Considering that climate change in the Aral Sea region is increasingly acute, the newly afforested areas need to be monitored for survivability in the absence of management (for instance, impact of water shortage, possible illegal logging, temperature fluctuations and so on) in the coming decades.

Alongside multiple obvious benefits of NbS, the literature states also a number of challenges associated with their understanding, implementation, effectiveness, stakeholder participation (Nelson, Bledsoe, Ferreira, & Nibbelink, 2020), lack of standards for their evaluation (Kumar, et al., 2021), (Fernandes & Guiomar, 2018). However, it is crucial to mention that the success of NbS and the long-term effectiveness of their socio-economic benefits highly depend on the environmental awareness (Gómez Martín, Máñez Costa, Egerer, & Schneider, 2021), and thus perception and understanding of NbS and their potential benefits.

The term “nature-based solutions” is relatively new, hence more evidence-based documentation of success stories around the world is needed to validate both cost-efficiency and long-term applicability of these practices, especially in those regions with newly formed ecosystems like the Aralkum.

## Appendix 1 Results of keyword search on NbS in dryland ecosystems

**Table:**

№	Keyword combination	Number of records in Scopus	Number of records in the Web of Science	Total number of non-duplicative records	Number of relevant articles read
1.	Nature-based AND solutions	1936	1646	2095	68
2.	Ecosystem-based AND drylands	10	7	10	4
3.	Nature-based AND solutions AND terrestrial	41	36	46	3
4.	Nature-based AND solutions AND desert	8	8	10	3
5.	Nature-based AND solutions AND heathland	1	1	1	1
6.	Nature-based AND solutions AND dryland	4	6	9	1
7.	Nature-based AND solutions AND wetland	194	199	251	19
8.	Nature-based AND solutions AND rangeland	2	2	2	1
9.	Nature-based AND solutions AND grassland	25	28	35	2
10.	Nature-based solutions AND protected area	48	84	98	4
**Total**		**2269**	**2017**	**2557**	**106**

## Appendix 2 Results of keyword search on NbS in the Aral Sea region

**Table:**

№	Keyword combination	Number of records in Scopus	Number of records in the Web of Science	Total number of non-duplicative records	Number of relevant articles read
1.	Ecosystem-based AND Aral Sea	2	2	2	1
2.	Nature-based AND solutions AND Central Asia	4	5	6	1
3.	Nature-based AND solutions AND Aral Sea	1	2	2	1
4.	Rangeland AND Aral Sea	4	2	6	4
5.	Grassland AND Aral Sea	13	14	21	9
6.	Afforestation AND Aral Sea	19	24	30	6
7.	Artemia AND Aral Sea	9	7	9	4
8.	Wetlands AND Aral Sea	45	51	70	11
9.	Saxaul AND Aral Sea	4	3	5	3
**Total**		**101**	**110**	**151**	**40**

## Footnotes

1 A new resolution of the Cabinet of Ministers of Uzbekistan was adopted (effective of 19 January 2022) to increase “forest plantations” on the Aral Seabed.

2 (Decree of the Cabinet of Ministers of the Republic of Uzbekistan on Additional measures for the creation of forests in the regions and of the “green cover” in the Aral Sea region, 2020b)

3 (UNEP-WCMC, 2022a)

4 (UNEP-WCMC, 2022b)

5 (Decree of the Cabinet of Ministers of the Republic of Uzbekistan on the establishment of South Ustyurt National Park (in Uzbek language), 2020a)

6 (UNEP-WCMC, 2023)

7 (Decree of the Cabinet of Ministers of the Republic of Uzbekistan on additional measures related to the organization of protected areas on forestlands, 2022)

8 My Garden in the Aral Sea – International innovation Centre for Aral Sea basin under the President of the Republic of Uzbekistan (iic-aralsea.org)

9 Home|#GreenAralSea

10 FAO classification of soil types (Schad, 2014)
